# Perturbation of synapsins homeostasis through HIV-1 Tat-mediated suppression of BAG3 in primary neuronal cells

**DOI:** 10.1038/s41419-019-1702-2

**Published:** 2019-06-17

**Authors:** Taha Mohseni Ahooyi, Bahareh Torkzaban, Masoud Shekarabi, Farzaneh G. Tahrir, Emilie A. Decoppet, Bianca Cotto, Dianne Langford, Shohreh Amini, Kamel Khalili

**Affiliations:** Department of Neuroscience Center for Neurovirology, Lewis Katz School of Medicine at Temple University, 3500N. Broad Street, Philadelphia, PA 19140 USA

**Keywords:** Molecular biology, Neuroscience, Diseases of the nervous system, Infectious diseases

## Abstract

HIV-1 Tat is known to be released by HIV infected non-neuronal cells in the brain, and after entering neurons, compromises brain homeostasis by impairing pro-survival pathways, thus contributing to the development of HIV-associated CNS disorders commonly observed in individuals living with HIV. Here, we demonstrate that synapsins, phosphoproteins that are predominantly expressed in neuronal cells and play a vital role in modulating neurotransmitter release at the pre-synaptic terminal, and neuronal differentiation become targets for Tat through autophagy and protein quality control pathways. We demonstrate that the presence of Tat in neurons results in downregulation of BAG3, a co-chaperone for heat shock proteins (Hsp70/Hsc70) that is implicated in protein quality control (PQC) processes by eliminating mis-folded and damaged proteins, and selective macroautophagy. Our results show that treatment of cells with Tat or suppression of BAG3 expression by siRNA in neuronal cells disturbs subcellular distribution of synapsins and synaptotagmin 1 (Syt1) leading to their accumulation in the neuronal soma and along axons in a punctate pattern, rather than being properly distributed at axon-terminals. Further, our results revealed that synapsins partially lost their stability and their removal via lysosomal autophagy was noticeably impaired in cells with low levels of BAG3. The observed impairment of lysosomal autophagy, under this condition, is likely caused by cells losing their ability to process LC3-I to LC3-II, in part due to a decrease in the ATG5 levels upon BAG3 knockdown. These observations ascribe a new function for BAG3 in controlling synaptic communications and illuminate a new downstream target for Tat to elicit its pathogenic effect in impacting neuronal cell function and behavior.

## Introduction

Despite the considerable advances in increasing life spans of people living with HIV, after treatment with ART, HIV-associated neurological disorders (HAND) remain a comorbidity that affects a large number of HIV-1 positive patients^1^. Although HIV-1 does not infect neurons, it promotes neurodegenerative processes through release of HIV-1 neurotoxic proteins, such as Nef and Tat, from infected bystander cells that subsequently undergo endocytosis by neurons^2^. Tat is a known neurotoxic agent, as it alters bioenergetics and survival pathways of neurons like cholesterol homeostasis^3^ and leads to alterations in dopamine secretion^4^ and synaptic loss^5^. Furthermore, Tat has been shown to reduce neuronal excitability, damage dendritic spines^6^ and potentiate inhibitory GABA_A_ receptors^7^. It also affects synaptodendritic stability^8^ and induces cell death and synapse loss through NMDA (N‐methyl‐d‐aspartate) receptor activation^9^.

Synapsins, including Syn I, Syn II, Syn III, and their isoforms, are phosphoproteins that are predominantly expressed in neurons with diverse functions ranging from synaptic vesicle transport and neurotransmitter release to synaptogenesis. Altered expression and post-translational modifications (e.g., hyper-phosphorylation) of synapsins have been implicated in Alzheimer’s disease (AD)^10–14^, Parkinson’s disease (PD)^15–17^, Huntington’s disease (HD)^18^, schizophrenia^19–22^, autism spectrum disorders (ASD)^23–26^, and others. In this context, aggregation of synapsins has been observed in various neurodegenerative diseases^26^. Functional dysregulation and turnover of synapsins have also been shown to lead to protein aggregation and deposition of proteins such as α-synuclein fibrils^15^ and amyloid beta^12^.

BAG3 is a stress induced cellular protein that has recently received much attention due to its role in regulating apoptosis and autophagy^27–29^. It is well-established that BAG3 is involved in protein quality control (PQC) and its activation during stress, viral infection, and mechanical insult facilitates removal of the damaged, mutated and truncated proteins from the cell environment^30^. BAG3 downregulation coincides with loss of mitochondrial membrane potential^31^, caspase activation, apoptotic DNA fragmentation and autophagy impairments^32^. Interactions of BAG3 with Hsp70 and Bcl2 provide it with properties to regulate apoptosis, whereby Hsp70 interferes with cytochrome c release^33^. BAG3 has been shown to activate lysosomal autophagy and promote higher turnover of small heat shock proteins (sHsps)^34^.

BAG3 contains multiple domains mediating its interactions with different families of proteins. The BAG domain, IPV motif and PXXP domain assist in its binding to Hsp70, small heat shock proteins and dynein, respectively. These interactions result in anti-apoptotic activity, selective-autophagy and macro-autophagy, and aging related alterations. BAG3 also utilizes its WW domain to communicate with SYNPO2 and TAZ/YAP signaling to induce gene transcription while modulating autophagy and autophagosome association and an increased formation of LC3-II^35^. BAG3 regulates viral replication, and is involved in the host response^36^. For example, BAG3 suppresses HIV-1 gene expression through inhibiting the interaction of NFκB with the DNA motifs within the integrated viral genome^37^.

In the brain, BAG3 is involved in the degradation of alpha synuclein (α-Syn), an aggregate forming protein implicated in Parkinson’s disease^38^, and also co-localizes with tyrosine hydroxylase (TH) in brain^38^. BAG3 interacts with a wide variety of aggregate forming peptides common among neurodegenerative diseases including α-Syn, polyglutamine (PolyQ) and tau, among others^39^. Both BAG3 protein and mRNA have been detected in synaptosomal polysomes, indicating its role in synaptic events such as plasticity^40^. In neuronal progenitor cells, mitogens FGF2 and EGF function as BAG3 transcription factors^41^. Alterations in the Hsp70/BAG3/HspB8 complex have been implicated in the accumulation of misfolded proteins and emergence of motor neuron diseases such as amyotrophic lateral sclerosis (ALS) and distal motor neuropathy^42–45^. The involvement of BAG3 in selective macroautophagy-mediated degradation of tau and polyQ, contributes to the emergence and progression of neurodegenerative diseases such as AD and HD^43–47^. In the brain, in addition to full-length BAG3, a shorter form of this protein (40 kD) has been detected in association with synaptosomes^46^. Earlier studies demonstrated that the absence of a transcription factor regulating cell response to oxidative stress, Nrf2, downregulated BAG3 in aged animals, leading to a lower rate of autophagy and an increased level of tau phosphorylation, indicating a potential involvement in tauopathies such as AD^48^. BAG3 has also been observed to be associated with chaperonin complex and actin folding^49^. Upon proteasome inhibition, BAG3 is modulated by cleavage of soluble products of amyloid precursor protein (APP)^50^. A BAG3 mutation has been linked to giant axonal neuropathy (GAN)^30^. In neurons, the location of BAG3 during development is different than in adult neurons. During development, BAG3 is localized at the sites of neurite outgrowth; whereas, in adult neurons, it is mostly located in the cytoplasm and peri-nuclear regions. This phenomenon points to a role for BAG3 during neuronal network development and maintenance. This shift of localization during neuronal differentiation and development is not accompanied with significant changes in the level of BAG3 protein where it is present in axon terminals and along processes^51^.

In the current work, we studied the effects of HIV-1 Tat on lysosomal autophagy, a major stress-induced PQC pathway in neurons, and the role of a key molecular regulator of chaperone activity, BAG3, on proteostasis in the context of turnover of markers of synaptic vesicles (SVs) such as synapsins and syanptotagmin 1 (Syt1). Our biochemical assays clearly indicated the detrimental effect of HIV-1 Tat on the normal distribution of synapsins essential to neuronal signal transmission. Central to this phenomenon, we discovered that levels of BAG3 mRNA and protein were significantly suppressed in the presence of Tat, further indicating downregulation of ATG5 protein and LC3-I lipidation to LC3-II, crucial steps in the cellular autophagy response to oxidative stress induced by Tat. This cascade of events led to the accumulation of synapsin positive puncta in the neuronal soma and processes proximal to it. Besides this autophagy-derived phenomenon, we confirmed the interaction of BAG3 with synaptic vesicle proteins, which probably serves a dual function: to transport SV proteins along axons and to sequester the aberrant synapsins and Syt1 through macroautophagy.

## Results

### Neuronal synaptic network is disrupted in the presence of HIV-1 Tat

**Fig. 1:**
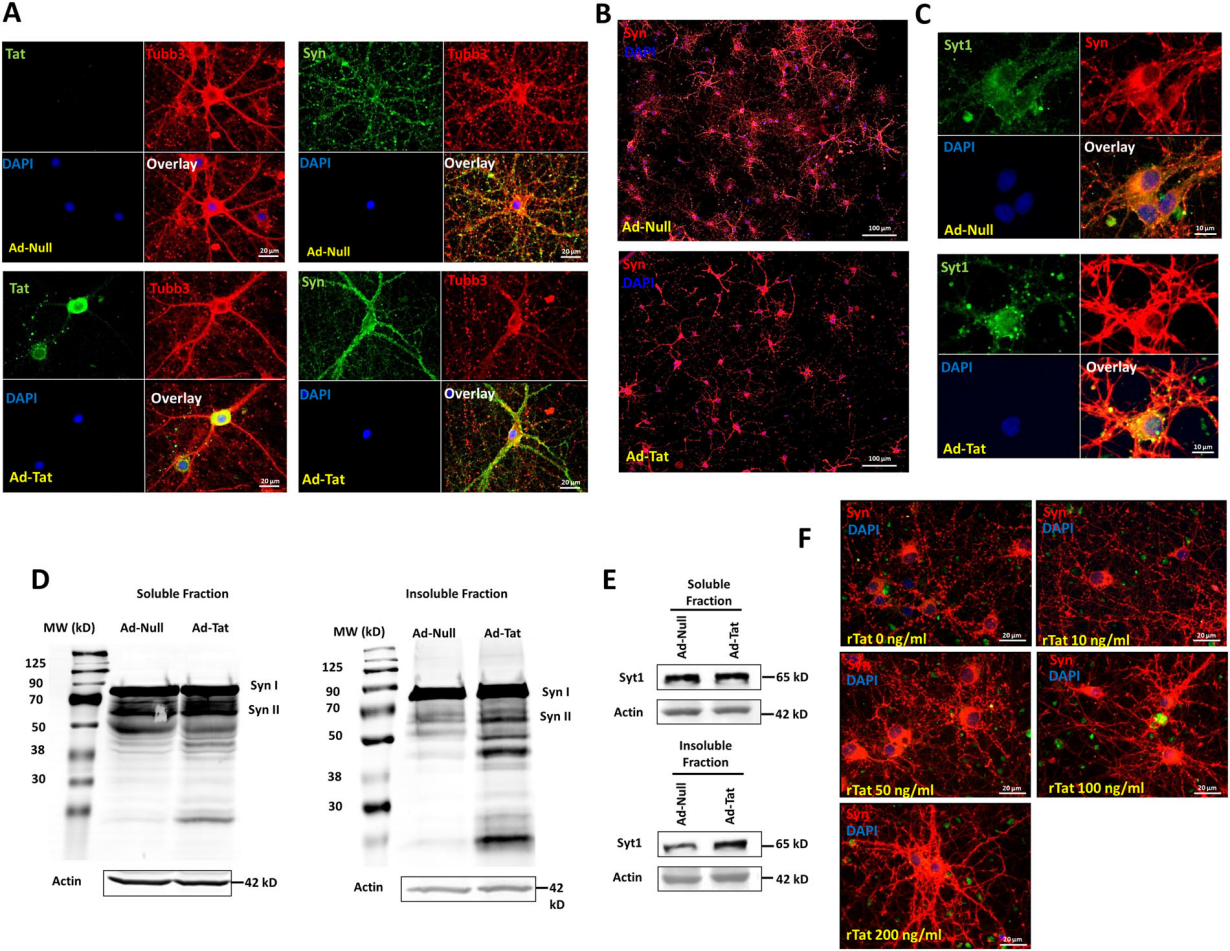
HIV-1 Tat promotes alterations in synaptic vesicle proteins distribution and homeostasis. **a** Single neuron images showing that Tat overexpression affects neuronal processes and synaptic vesicle distribution as stained with Tubb3 and synapsins (Syn), respectively. Synapsins accumulate close to soma upon Tat expression, compared to the control where synapsins are primarily observed in neuronal processes. **b** Synapsin distribution in neurons in a population wide image shows the disruption of synaptic vesicle network during Tat expression. **c** A second synaptic vesicle marker, synaptotagmin 1 (Syt1), confirms the aggregation of synaptic vesicle proteins in neurons upon Tat expression as compared to the Ad-Null transduction. **d** In addition to distribution, total synapsin proteins levels are altered in the soluble and insoluble fractions of neuronal lysate upon Tat expression. **e** Synaptotagmin 1 exhibits more accumulation in the insoluble fraction compared to soluble fraction (~70%) in neurons where Tat is expressed. **f** Dose response of soluble recombinant Tat (rTat) protein on the distribution of synapsins indicates the increased impairment at higher doses of Tat. Syt1, Syn, and Tubb3 stand for synaptotagmin 1, synapsins, and β3-tubulin, respectively

Transduction of HIV-1 Tat protein via an adenoviral vector significantly alters cellular distribution of synapsins. Our immunocytochemical labeling of synaptic vesicles in rat hippocampal neurons shows that Tat expression leads to accumulation of synapsins positive puncta close to the soma rather than more widespread distribution throughout the neuronal axon terminals (Fig. 1a, b). Synaptotagmin I, another neuronal synaptic vesicle marker, is similarly located close to the the soma upon expression of Tat (Fig. 1c). Further investigation of the synaptic vesicle proteins, synapsins and Syt1 using immunoblotting indicated that Tat not only alters synaptic distribution, but the proteins present in different cellular compartments (Fig. 1d, e). After fractionation of soluble (cytosolic) and insoluble (membrane, nuclei-related and aggregates) protein extracts, we probed for synapsins and Syt1 (Fig. 1d, e). In both cases, a significant increase (over 70%) in protein levels was observed in the insoluble fraction upon Tat expression, suggesting that Tat promotes formation of synaptic protein aggregates. To assess the dose dependent impact of Tat on synapsins-positive aggregates and subcellular localization, hippocampal neurons were exposed to different concentrations of recombinant Tat (rTat) protein. Results indicated that with increasing Tat concentration, increased synapsin-positive aggregates proximal to the neuronal soma were observed (Fig. 1f). This aggregation appears in the form of puncta in the cell body and processes proximal to soma (Fig. 1c, f).

### Tat downregulates BAG3 protein levels by decreasing its mRNA levels

**Fig. 2:**
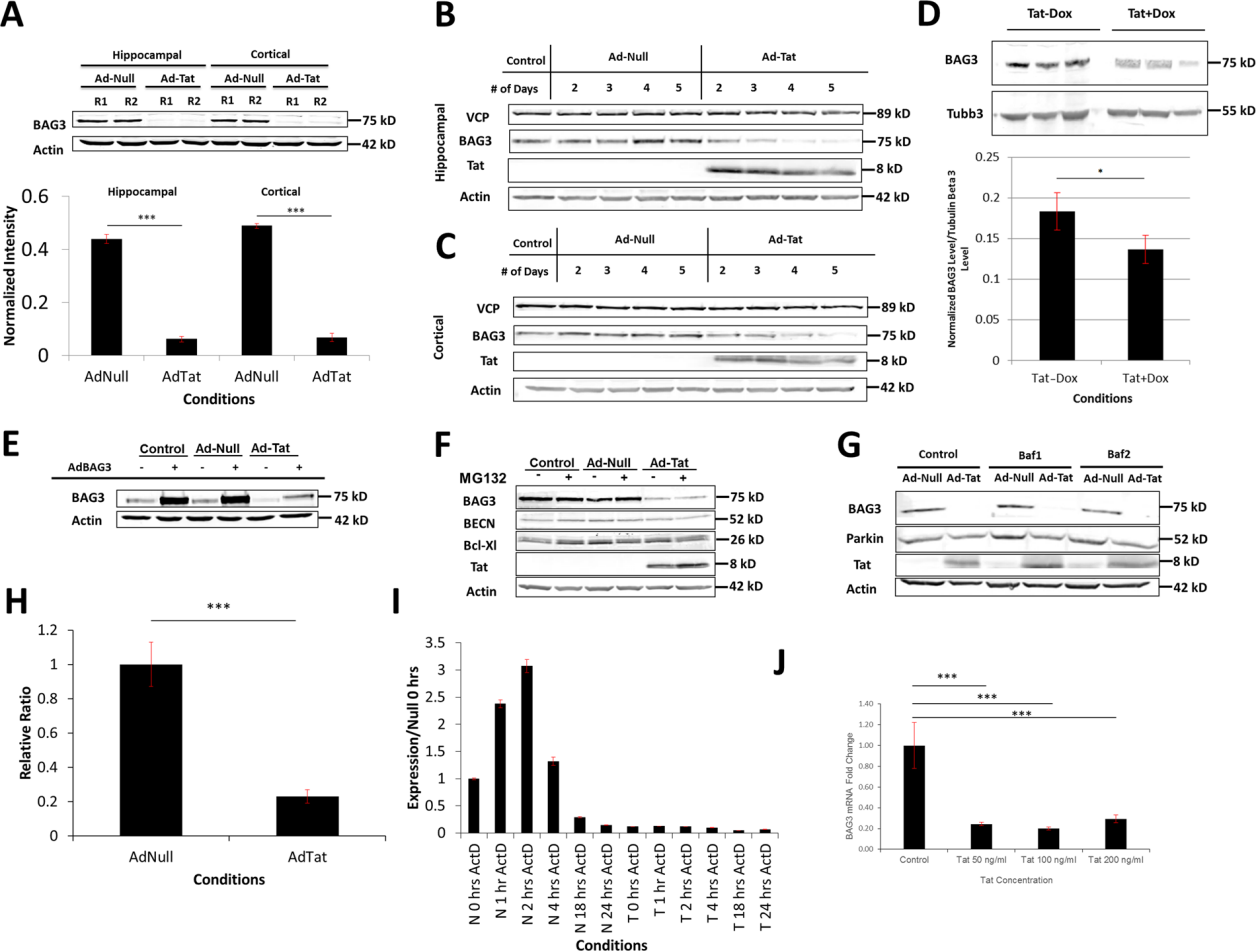
HIV-1 Tat decreases BAG3 protein and mRNA. **a** Tat expression reduced BAG3 protein in rat primary hippocampal and cortical neurons, R1 and R2 indicate two replicates shown on this gel. **b**, **c** Hippocampal and cortical neurons expressing Tat show time-dependent reduction in BAG3 (VCP was probed as a control). **d** BAG3 level drops in the brains of Tat-transgenic mice under Dox-induced Tat expression. **e** Tat expression results in a decreased level of BAG3 protein even during adenoviral overexpression of BAG3, indicating a post-transcriptional reduction. **f** Inhibition of the proteasome using MG132 does not restore Tat-induced BAG3 reduction. **g** Inhibition of the lysosome using Baf A1 does not block Tat-induced BAG3 decrease. **h** Neuronal BAG3 mRNA is decreased by Tat expression. **i** qRT-PCR measurement of BAG3 mRNA levels under control and Tat expression when transcription is inhibited by Actinomycin D. **j** Recombinant Tat decreases BAG3 mRNA levels. Quantifications show mean ± SD with *n* ≥ 3. *P*-values calculated using *t*-test where *P ≤ 0.01, **P ≤ 0.005, ***P ≤ 0.001

Tat expression significantly suppressed BAG3 protein levels in both hippocampal and cortical neurons (Fig. 2a). Time course studies of Tat expression revealed that Tat gradually diminishes BAG3 in the whole-cell extract in neurons. This phenomenon was observed starting 48 h post-transduction and continued until complete disappearance of BAG3 around day 6 post-transduction (Fig. 2b, c). Similarly, DOX-induced Tat expression in the brains of Tat-transgenic mice, led to a decrease in BAG3 in the whole brain protein lysate (Fig. 2d). Tat expression also blocked the adenoviral overexpression of BAG3 (Fig. 2e). To determine the mechanism underlying the Tat-mediated reduction of BAG3, a series of experiments were conducted to assess rates of transcription and translation. We first hypothesized that Tat might cause BAG3 degradation through different proteolysis pathways. To that end, the two major protein degradation pathways, proteosomal and lysosomal pathways were separately blocked using appropriate inhibitors. MG132 was used to inhibit the proteasome pathway for 4 h on day 3 post-transduction. Since BAG3 levels were not altered upon MG132 application and thus proteasome inhibition, the role of this degradation pathway was ruled out (Fig. 2f). A second major protein degradation pathway was examined by application of Bafilomycin A1 (Baf A1) to inhibit lysosomal degradation. Once again, no significant changes in BAG3 levels were observed, suggesting lysosomal degradation is unlikely to be involved in Tat-mediated BAG3 decline further implicating transcriptional or post transcriptional regulation (Fig. 2g). To examine this hypothesis, we assessed BAG3 mRNA levels in the presence and absence of Tat expression in hippocampal neurons. In agreement with our previous data, Tat downregulated BAG3 mRNA (Fig. 2h). To examine the stability of BAG3 mRNA, we blocked transcription with Actinomycin D and quantified BAG3 mRNA levels in control and Tat expressing neurons (Fig. 2i). The results suggested that the BAG3 mRNA half-life, despite the multiple fold lower basal level under Tat expression, did not change significantly (data not shown). Finally, we treated neurons with of various concentrations of rTat protein (50, 100, and 200 ng/ml) and observed the same inhibitory effect on BAG3 mRNA (Fig. 2j).

### BAG3 suppression promotes formation of aggregates containing synaptic vesicle proteins

**Fig. 3:**
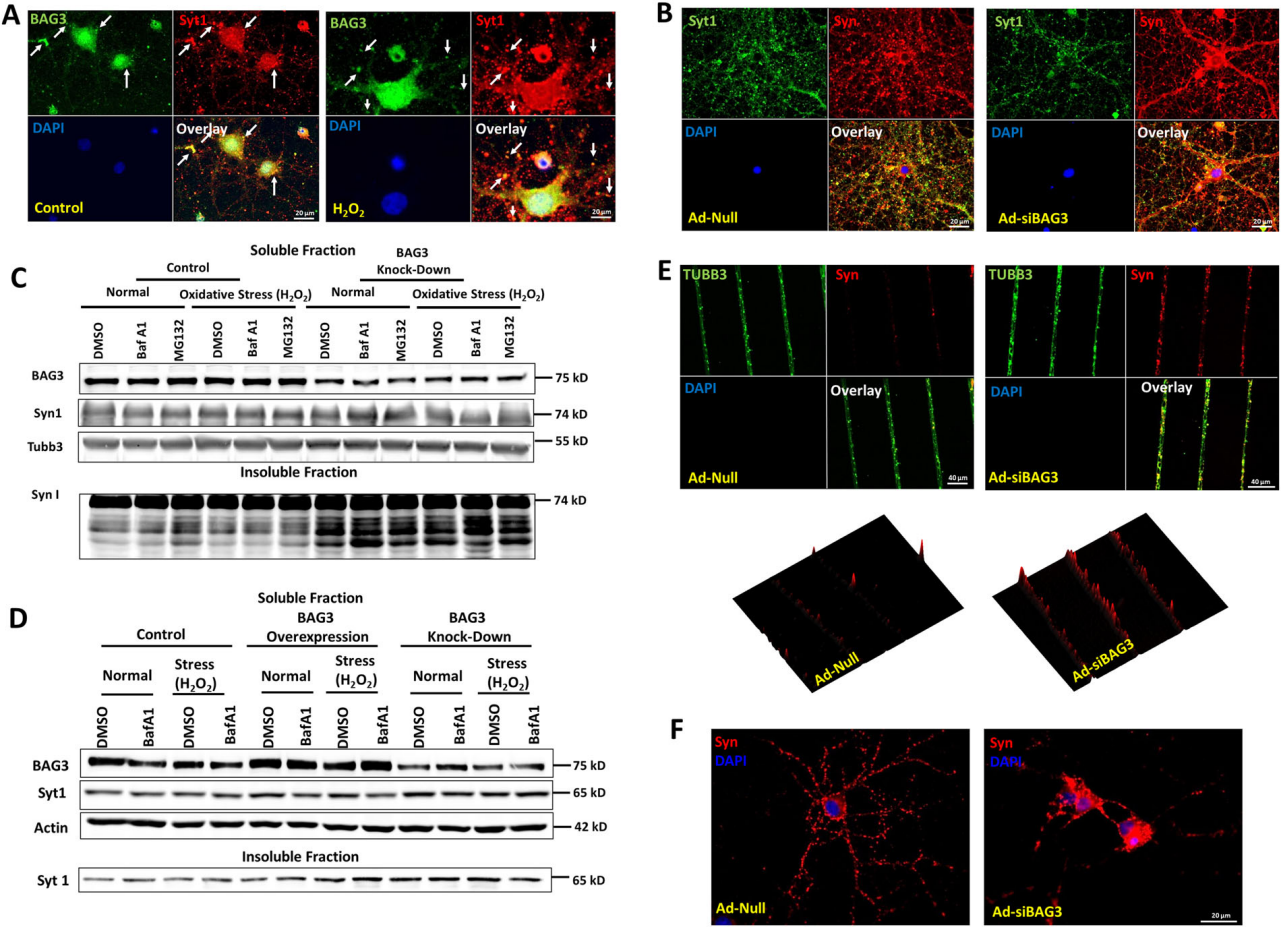
BAG3 KD leads to the accumulation and aggregation of synaptic vesicle proteins. **a** BAG3 co-localizes with Syt1 in the control and oxidative-stress induced neurons. **b** SVs probed with Syn and Syt1 indicate the accumulation of synaptic vesicles along the major neuronal processes and soma under BAG3 KD. **c** BAG3 KD under normal and H_2_O_2_-induced oxidative stress conditions results in accumulation of Syn in the insoluble fraction of neuronal protein lysate. **d** BAG3 KD under normal and stress conditions results in accumulation of Syt1 in the insoluble fraction of neuronal protein lysate. **e** Neuronal culture in a microfluidic device allows the assessment of neuronal processes separate from the soma. BAG3 KD using Ad-siBAG3 transduction leads to the increased formation and accumulation of syn-positive aggregates along processes. **f** BAG3 KD impairs the distribution of synapsins in the neurons as compared to the control

As observed with Tat-mediated localization of synapsin-positive puncta to the neuronal soma, siRNA-mediated BAG3 knockdown had a similar effect on synapsin distribution by promoting its accumulation in the soma proximity (Fig. 3a, b). Further, western blot analysis following BAG3 knockdown (KD) demonstrated an aggregation of Syn and Syt1 in the insoluble fraction (Fig. 3c, d). Accumulation of Syt 1 appeared to be affected more by the BAG3 KD compared to the autophagy inhibition. This phenomenon could be because of the longer half-life of the protein compared to the duration of the Baf A1 treatment. The aggregation of synapsin-positive puncta under BAG3 knockdown was further investigated using a microfluidic device generating micrometer width grooves. Neurons were plated on a built-in set of wells and processes extended along the grooves to allow for assessment of axonal aggregation of synapsins. Knockdown of BAG3 led to the formation of large aggregates within the axons (Fig. 3e, f and S3C), as compared to control. Quantification of the aggregated synapsins revealed that BAG3 KD resulted in the formation of 1.9-fold as many synapsin-positive aggregates compared to the control (Fig. 3e, bottom row). Finally, adenoviral-mediated expression of HIV-1 Tat in cortical neurons resulted in the accumulation of aggregated Syt1 in the insoluble proteins fraction under control and lysosomal autophagy inhibition (Fig. S1B).

### HIV-1 Tat and BAG3 suppression downregulate ATG5 and inhibit lysosomal autophagy

**Fig. 4:**
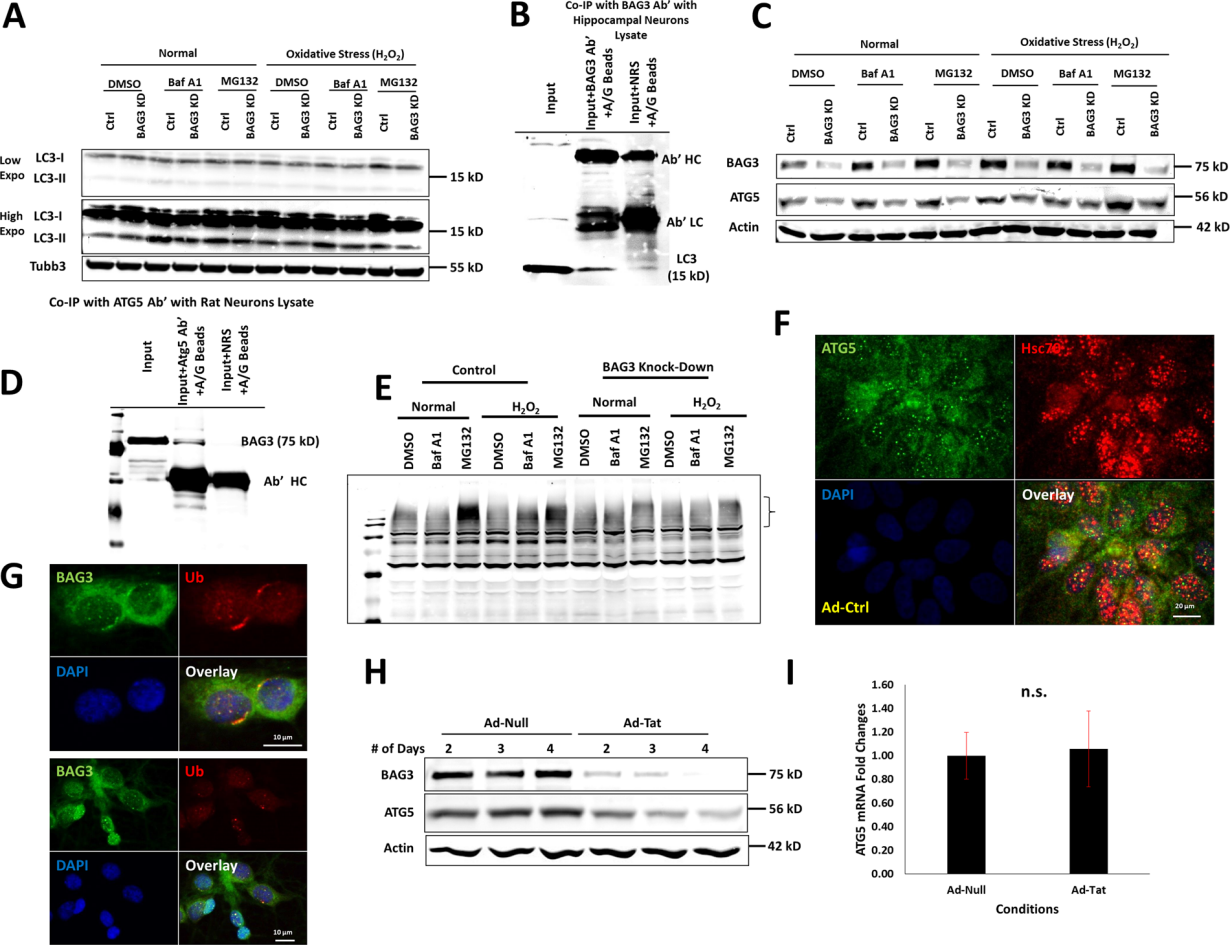
BAG3 KD impairs lysosomal autophagy and client protein ubiquitination under oxidative stress. **a** Under control and oxidative stress conditions, BAG3 KD leads to decreased level of LC3-II. **b** Co-immunoprecipitation with BAG3 and LC3 confirms the interaction of BAG3 and LC3. **c** Under control and oxidative stress conditions, BAG3 KD leads to reduced levels of ATG5, a protein essential for LC3-I to LC3-II conversion. **d** Co-immunoprecipitation with ATG5 antibody and probing for BAG3 confirms the interaction of BAG3 and ATG5. **e** BAG3 KD in neurons leads to inhibition of ubiquitination under oxidative stress and inhibition of proteasome or lysosome. The bracket shows the higher molecular weight region at which ubiquitination is impaired under BAG3 KD. **f** ATG5 co-localizes with Hsc70 in neurons. **g** BAG3 co-localizes with UB in neurons. **h** ATG5 and BAG3 are suppressed under Tat expression over a range of 2–4 days post-transduction. **i** ATG5 mRNA level is not decreased by Tat expression compared to control

To investigate the possible mechanisms underlying SV proteins aggregate formation under BAG3 KD and Tat expression, we studied the lysosomal protein degradation pathway under BAG3 KD and oxidative stress to simulate the effects of Tat. BAG3 KD led to a significant reduction in LC3-II formation and autophagy flux under the control and H_2_O_2_-induced oxidative stress conditions (Fig. 4a). Co-immunoprecipitation suggested an association between BAG3 and LC3 (Fig. 4b) and BAG3 and ATG5 (Fig. 4d), an essential protein for LC3-I lipidation and conversion to LC3-II is required for autophagosome formation. Our data indicated that BAG3 KD, under proteasome and lysosome inhibition and during oxidative stress, led to ATG5 downregulation (Figs. 4c and S3). BAG3 KD adversely affected ubiquitinated protein levels under oxidative stress; while the presence of oxidative stress shift the ubiquitination flux from the proteasome to lysosome under the control neurons, BAG3 KD impairs this shift (Fig. 4e). ATG5 co-localized with Hsc70, a canonical heat shock protein and a BAG3 partner, as determined by immunocytochemistry imaging (Fig. 4f). BAG3 was also shown to co-localize with ubiquitin and/or ubiquitinated proteins (Fig. 4g). Finally, Tat expression downregulated ATG5 levels in a time-dependent manner (Fig. 4h), with no effect on its mRNA levels (Fig. 4i).

### BAG3 interacts with synaptic vesicle proteins

**Fig. 5:**
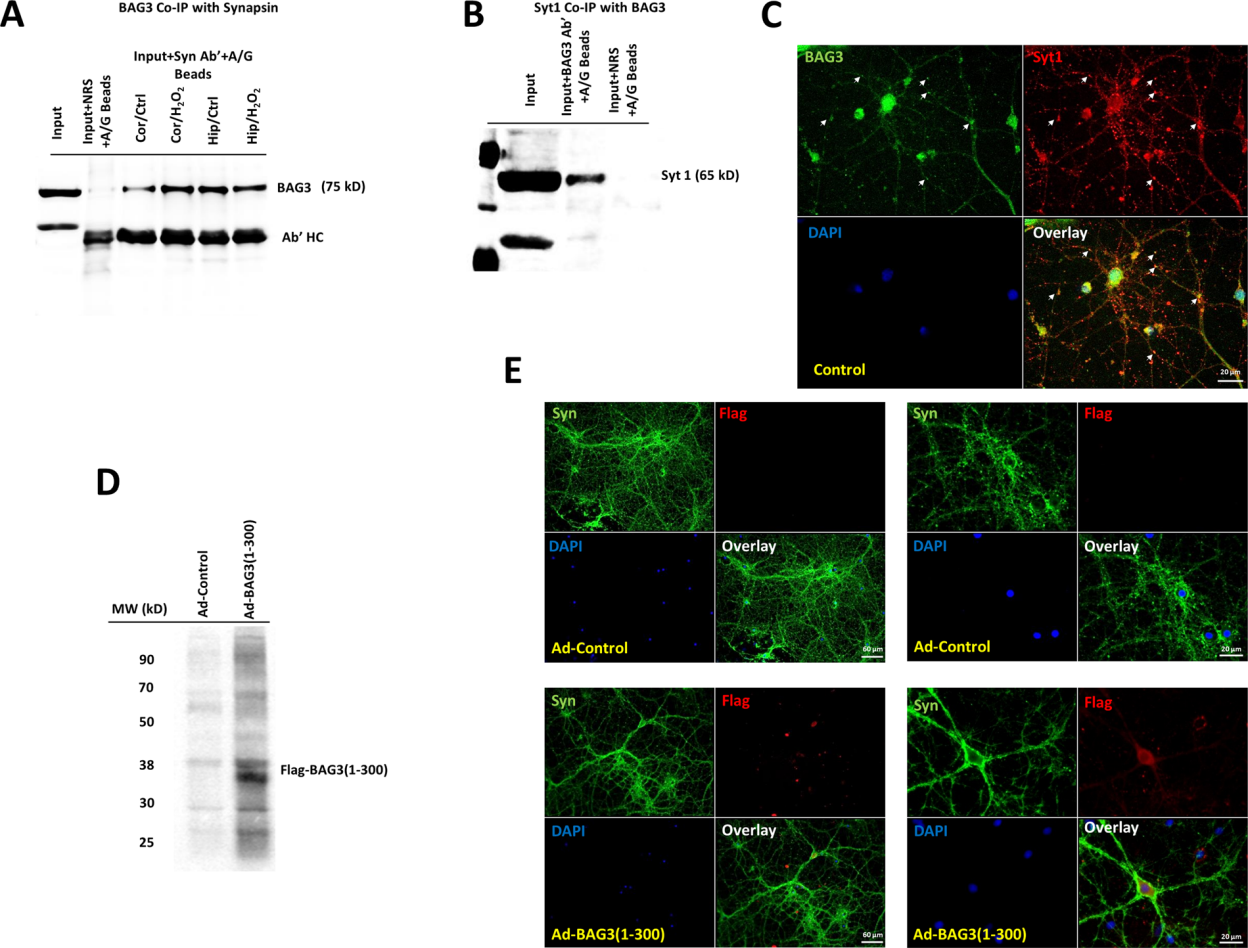
BAG3 interacts with synaptic vesicle proteins and synapsins likely via the WW domain. **a** Co-immunoprecipitation with Syn antibody and probing for BAG3 confirms the interaction of BAG3 with synapsins in rat primary cortical and hippocampal neurons under normal and oxidative stress conditions. **b** Co-immunoprecipitation with BAG3 antibody and probing for synaptotagmin 1 confirms the interaction of BAG3 and Syt1. **c** BAG3 co localizes with Syt1 in neurons. **d**, **e** Expression of Flag-BAG3 (1–300) in neurons results in the disruption of neuronal synapsins distribution similar to BAG3 KD and Tat expression

We next examined whether BAG3 interacts with synaptic vesicle proteins including synapsins and Syt1 by utilizing different biochemical assays. Co-immunoprecipitation with synapsins antibody and probing for BAG3 clearly indicated BAG3 interaction with synapsins in both hippocampal and cortical neurons, under both normal and H_2_O_2_-induced oxidative stress (Fig. 5a). co-immunoprecipitation with BAG3 antibody resulted in pulldown of synaptotagmin 1 (Fig. 5b). As depicted in Fig. 5c, BAG3 co-localizes with Syt1 in rat hippocampal neurons. To examine whether there is an interaction between BAG3 WW domain and proline-rich domains of synapsins and if this would affect synaptic vesicle proteins distributions, we overexpressed a BAG3 mutant containing only the WW domain and the IPV motif (BAG3 1–300) using an adenovirus vector. As shown in Fig. 5d, e, overexpression of the WW domain without the PXXP domain of BAG3, which mediates its interaction with motor protein dynein, disrupts the normal synapsins distribution and leads to a similar effect as observed in BAG3 KD and Tat expression.

## Discussion

**Fig. 6:**
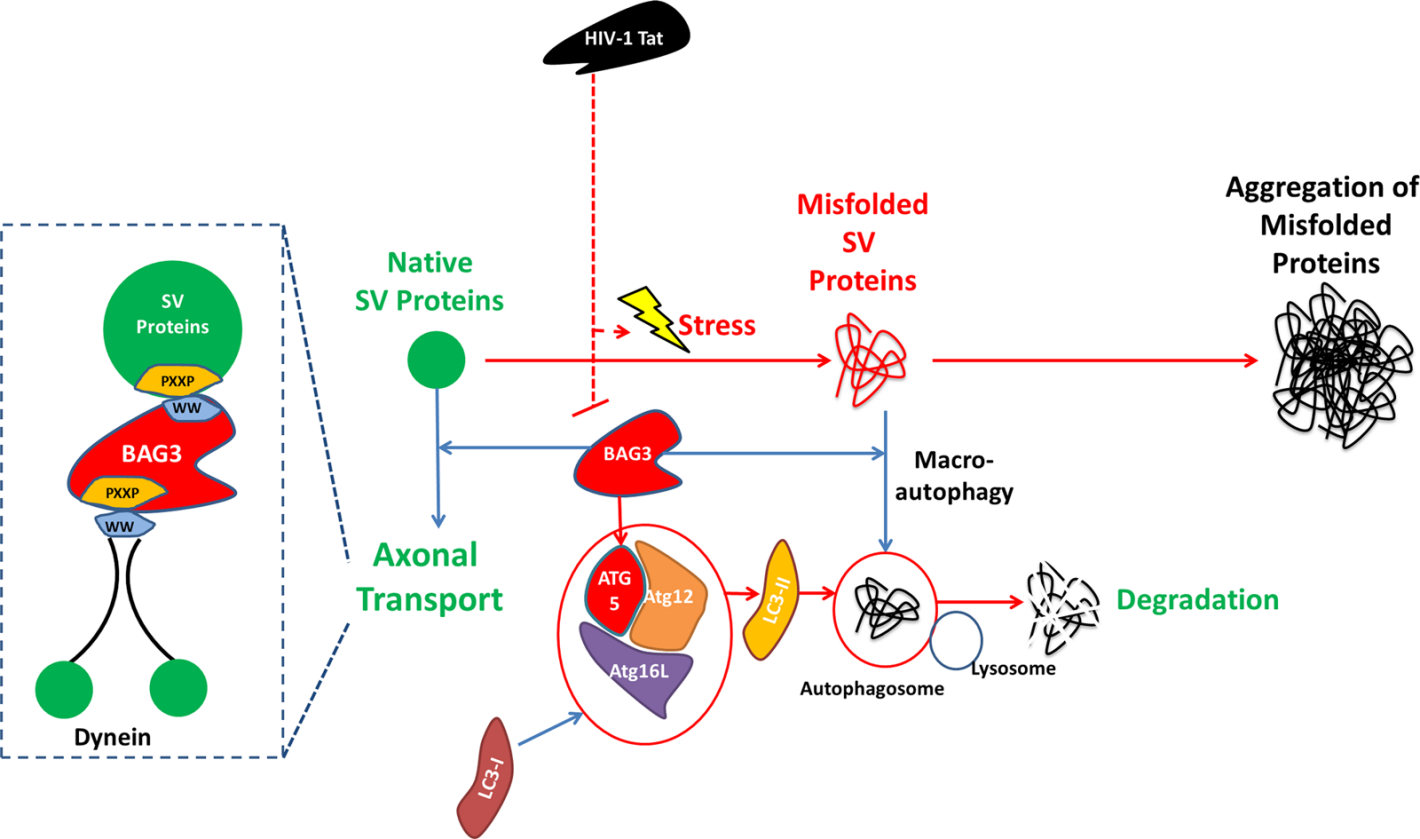
BAG3-mediated homeostasis of synaptic vesicle proteins turnover disrupted by HIV-1 Tat. BAG3 is essential for the formation of LC3-II via regulating ATG5 protein. BAG3 is involved in the homeostasis and turnover of synapsins through lysosmal autophagy and vesicle axonal transport

In this study, we showed that HIV-1 Tat-induced alterations in synaptic vesicle proteins, namely synapsins and synaptotagmin 1. These effects influenced not only the distribution of these proteins in neurons but also the total levels of synapsin protein family members in the soluble and insoluble protein fractions of neuronal cell lysate. Recently, it has been shown that synaptic vesicles were transported along axons via fast and slow axonal transport mechanisms and could involve Hsc70 (a member of Hsp70 family) chaperone activity^52,53^. BAG3 is a regulator of Hsp70 chaperone activity through utilizing motor protein dynein to direct degradable substrates to aggresomes in an ubiquitin-independent manner^54^. Conversely, inhibition of dynein-dependent retrograde transport of aggregated proteins to perinucleus aggresomes diverted neurons to activate proteasomal autophagy and reduced protein aggregates in a BAG1 dependent manner with implications in proteinopathy in motoneuron diseases^55^. We performed a comprehensive set of experiments to study the effects of HIV-1 Tat on BAG3. Our data clearly demonstrate the negative impact of Tat on BAG3 homeostasis in neuronal cells, both in dissociated rat cortical and hippocampal neurons and in Tat-transgenic mice and Tg26 mice brain slices. The inhibitory effect of Tat on BAG3 was not restored by interfering with lysosomal and proteasomal autophagy pathways, suggesting that Tat did not affect BAG3 through protein destabilization. Our qRT-PCR data showed that Tat expression using an adenoviral vector as well as treatment of neurons with recombinant Tat reduced the BAG3 mRNA level. Tat application together with overexpression of BAG3 using a different promoter (CMV) also led to reduced levels of BAG3 protein. These observations suggest that Tat suppresses BAG3 through a post-transcriptional effect on BAG3 mRNA. To examine the potential role of BAG3 in the homeostasis of synapsins, we knocked down BAG3 using a BAG3 siRNA. Similar to what was observed following Tat treatment, BAG3 KD led to the accumulation of synapsins in the proximity neuronal soma and aggregation of synapsins along axons grown in the microgroove device. Both synapsins and another synaptic vesicle marker, synaptotagmin 1, were shown to accumulate in the insoluble fraction of cell lysate following BAG3 KD. This phenomenon is hypothesized to partially occur through impairment of degradation of obsolete synaptic proteins and/or impairment of synaptic vesicle proteins axonal transport mediated by direct interaction with BAG3. Through its multi-domain structure, BAG3 is engaged in diverse proteome homeostasis processes. These complexes can target proteins for degradation, ubiquitination and further transport them along the microtubule towards aggresome formation. SQSTM1 helps BAG3-associated protein aggregates be engulfed by phagophore through binding to LC3^38^. BAG3 overexpression has been observed along with heat and mechanical stress that activate chaperone assisted autophagy mechanisms^56^. This process can be induced by inhibiting proteasome, which consequently leads to the activation of BAG3-dependent lysosomal autophagy and LC3-I to LC3-II conversion^29^. BAG3 depletion results in reduced levels of LC3-II and lysosomal autophagy^47^. LC3-II is reduced upon BAG3 KD; when ATG5 is knocked-down, BAG3 overexpression cannot restore LC3-II^38^ levels. In accordance with these earlier observations, we showed an interaction between BAG3 with both LC3 and ATG5. Furthermore, we demonstrated that BAG3 KD led to decreased levels of ATG5 and LC3-II formation under normal conditions and oxidative stress. Our results indicated co-localization of BAG3 with ubiquitinated aggregates, possibly aggresomes, in the neuronal soma. More interestingly, following BAG3 KD, it appeared that neuronal ubiquitination machinery did not respond to oxidative stress. Furthermore, Tat expression reduced the level of ATG5 in a time dependent manner, similar to BAG3 suppression (Fig. 4). Collectively, it can be concluded that BAG3 is essential for autophagy of ubiquitinated proteins in general and the LC3-II lipidation and lysosomal autophagy through maintaining the levels of ATG5 protein, in particular (ATG5 mRNA was not affected by BAG3 KD or Tat expression). To examine the interaction of BAG3 and synaptic vesicle proteins, we performed co-immunoprecipitation assays using BAG3 and Syn antibodies and probed with Syt1 and BAG3 antibodies, respectively. In both cases, the interaction of BAG3 and synaptic vesicle proteins was confirmed (Fig. 5). BAG3 also co-localized with synaptotagmin 1 in immunocytochemistry assay. BAG3 PXXP domain helps it bind to dynein, giving it vesicle transport activity of SH3 domain containing proteins including signal transducing adapter proteins and a broad range of protein kinases. WW domain of BAG3 mediates interactions with PXXP domain of other proteins^57^ and possibly BAG3–BAG3 communication^58^. BAG3, in turn, utilizes its WW domain to interact with the proline rich domain (e.g., PPPY and PPSY) of the target proteins^56^. As all synapsin proteins possess a proline-rich domain, we examined whether this domain interacted with the WW domain of BAG3, facilitating its transport via dynein retrograde axonal transport mechanism. To this end, we overexpressed a BAG3 (1–300) mutant containing only the WW domain (with no PXXP to interact with dynein) to study the effect on synaptic vesicles proteins as probed by synapsins. This overexpression resulted in comparable observations as in BAG3 KD and Tat expression. Moreover, we showed that BAG3 overexpression restores the effect of Tat on synaptic vesicle distribution as well as neuronal electrophysiological activity measured by microelectrode arrays upon BAG3 KD and Tat expression. In conclusion, we propose a mechanism depicted in Fig. 6, where BAG3 interacts with synapsins and is essential for the homeostasis of these proteins as well as healthy state of synaptic vesicle proteins. The BAG3 suppression imposed by HIV-1 Tat in neurons has detrimental effects on SV proteins distribution and possibly synapse formation. These observations have implications in synaptic loss and alterations in neuronal plasticity as a result of HIV-1 infection and Tat expression.

### Experimental procedures

#### Tissue and cell culture

Tissue preparation and cell cultures were performed according to the guidelines approved by Temple University Institutional Animal Care and Use Committee.

##### Tat transgenic mice

Doxycycline (DOX)-inducible GFAP promoter driven HIV-1 Tat transgenic mice were provided by the Comprehensive NeuroAIDS Center. Adult mice (18–28 g) were singly housed in a temperature (21–23°) and humidity-controlled vivarium with constant airflow on a reverse 12-h light/dark cycle (lights off at 09:00). Food and water were available ad libitum. Mice expressed the HIV-1 *tat* transgene (TAT) or their control littermates. Briefly, TAT mice conditionally-expressed the HIV-1 Tat_1–86_ protein in an astrocyte-specific manner under the control of a GFAP-driven Tet-on promoter which is activated in the presence of DOX. TAT mice received an 80 mg/kg intraperitoneal (i.p.) injection of doxycycline hyclate (DOX). After this 14-day period, all mice were euthanized and prepared for harvest of brain tissue for western analyses.

##### Tg26 mice

Tg26 HIV transgenic mice are a well-described mouse model that encode the entire pNL4–3 HIV-1 genome minus a segment of gag/pol genes. This homozygous mouse expresses HIV viral proteins such as Tat and Nef and exhibits neuropathology similar to HIV-associated symptoms such as neurocognitive disorders and cardiac problems^59^.

##### Primary rat neuronal culture

Dissociated cell cultures of hippocampal and cortical neurons were prepared using dissected E18 prenatal rat embryonic brains. After digestion in 0.25% trypsin solution, neurons were plated on tissue culture plates and slides pre-coated with poly-d-lysine (Sigma, St. Louis, MO) and laminin (Invitrogen 23017, USA). Cells were maintained 14 days prior to treatments. Neurons were transduced with Ad-Null (Vector Biolabs, Malvern, PA), Ad-Tat (made in-house), Ad-siBAG3 (Vector Biolabs, Malvern, PA) and Ad-BAG3 (Vector Biolabs, Malvern, PA) with MOI 1 to 4 or recombinant Tat protein (50 ng/ml, full length, 101 amino acids, Immunodiagnostics, MA). Tat bioactivity was verified by the LTR-luciferase assay (data not shown). MG132 (5 µM) and Bafilomycin A1 (50 nM, Sigma) were applied for 4 h prior to lysis to block proteasome and lysosomal autophagy, respectively.

### Immunoblotting

Neuronal and mouse brain tissue lysates were prepared using RIPA lysis buffer (whole cell extract), Tris-Triton (soluble fraction) or 2% SDS in PBS (insoluble fraction), all containing protease inhibitor cocktail (Sigma–Aldrich, St. Louis, MO). Protein concentration was measured using Bio-Rad protein assay reagent (Bio-Rad, Hercules, CA). SDS-polyacrylamide gels, 10–12%, and nitrocellulose membranes (LI-COR, Inc., Lincoln, NE) were used for electrophoretic protein separation and transfer, respectively. Membranes were blocked (overnight at 4 °C) in Odyssey (LI-COR) blocking buffer and incubated with primary and secondary (1 h at RT) antibodies. Membranes were scanned using an Odyssey1 CLx Imaging System (LI-COR, Inc.) The following primary antibodies were used for Western blotting: BAG3 (Proteintech, Rosemont, IL, 10599-1-AP), Synapsin (Cell Signaling, D12G5), Synaptotagmin I (Santa Cruz, sc-136480), Tat (NIH AIDS Reagent Program, Germantown,MD, R705), LC3 (Sigma, L8918), ATG5 (Abcam, 108327), β3-Tubulin (Sigma, T8578), Bcl-xl (Santa Cruz, sc-8392), β-Actin (Santa Cruz, sc-47778), VCP (Santa Cruz, sc-20799), Beclin-1 (Cell Signaling Technology, 3738), Parkin (Abcam, ab77924), Tomm20 (Abcam, ab199641).

### Immunocytochemistry

Intracellular Tat was probed in Ad-Tat transduced neurons through immunocytochemistry. Neurons were transduced with Ad-Tat and Ad-Null on 14 DIV with MOI 1. After 72 hr, the cells were fixed and permeabilized using −20 °C cooled acetone (Sigma). Following blocking with (1%) BSA in PBST, the neurons were labeled with the following antibodies (1:100): BAG3 (Proteintech, Rosemont, IL, 10599-1-AP), Synapsin (Cell Signaling, D12G5), Synaptotagmin I (Santa Cruz, sc-136480), β3-Tubulin (Sigma, T8578), ATG5 (Abcam, 108327), Hsc70 (Enzo, N27F3-4), Ubiquitin (Santa Cruz, sc-8017) and rabbit polyclonal Tat antibodies (NIH AIDS Reagent Program, Germantown, MD, R705). Alexa Fluor®secondary antibodies (ThermoFisherScientific, OR) and VECTASHIELD medium (Vector Laboratories, Burlingame, CA) were used for labeling and mounting, respectively. Images were prepared via Leica fluorescent microscope (Leica Microsystems, IL).

### Immunohistochemistry

Brain tissue samples of wild type and Tg26 transgenic mice were collected and frozen tissue sectioning was embedded based on standard protocol. The thickness of sections was 9 µm. Sections were probed with anti-BAG3 (1:250) and anti-β3-tubulin (1:500) antibodies. The fluorescent staining followed the same procedure as described for immunocytochemistry.

### Cell culture on microfluidic device

Rat E18 hippocampal neurons were plated on microfluidic devices (Xona Microfluidics, TCND500) placed on glass cover slips (Leica Biosystems) coated with poly-d-lysine and laminin as described above. Neurons 14 DIV were transduced with Ad-Null and Ad-siBAG3. After 72 h cells were fixed and permeabilized in −20 °C cooled acetone (Sigma). The rest of the steps were similar to immunocytochemistry for labeling and imaging with synapsins and β3-tubulin.

### RNA isolation and cDNA preparation

Following treatment, total RNA for each condition was extracted using the Trizol (ThermoFisher, Carlsbad, CA) extraction protocol followed by RNA cleaning protocol using Direct-zol™ RNA MiniPrep Plus (Zymo Research, CA, USA) following the manufacturer’s recommendations. cDNA synthesis on total RNA samples was performed with High Capacity cDNA Reverse Transcription Kit (Thermo Fisher Scientific) according to manufacturer’s protocol.

### Real-time quantitative RT-PCR (qRT-PCR)

Specific primers for the BAG3 transcriptome were designed considering the exon junction positions in the genomic DNA (BAG3 Forward primer: 5′-GGCCCTAAGGAAACTGCAT-3′; Reverse primer: 5′-GGGAATGGGAATGTAACCTG-3′). The specificity and efficiency of the primers were checked by RT-PCR using Q5 High-Fidelity PCR Kit (New England Biolabs). All qPCR reactions were performed with the LightCycler96^®^ (Roche) using the SYBR™ Green master mix (Applied Biosystems, ThermoFisher), according to manufacturer’s protocol. Relative quantity was normalized to Actin expression.

### Microelectrode array-based electrophysiology

Microelectrode arrays (MEAs) were used to perform electrophysiological recordings at 2 kHz on an MEA60 system (Multichannel systems, Germany) from hippocampal neurons undergoing control, BAG3 KD, BAG3 overexpression, and Tat expression starting from 25 DIV.

## Supplementary information

Supplemental Material
