# Supplementary material for: Perturbation of synapsins homeostasis through HIV-1 Tat-mediated suppression of BAG3 in primary neuronal cells
Source: Cell Death Dis. 2019 Jun 17;10(7):473. doi: 10.1038/s41419-019-1702-2 (PMC6572798; doi:10.1038/s41419-019-1702-2)
Supplement: Supplementary file 1 — Supplemental Material [file 41419_2019_1702_MOESM1_ESM.pdf]

**3/6/19**

# Perturbation of Synapsin Homeostasis through HIV-1 Tat-Mediated Suppression of BAG3 in Primary Neuronal Cells

## SUPPLEMENTARY MATERIALS

Taha Mohseni Ahooyi†, Bahareh Torkzaban, Masoud Shekarabi, Farzaneh G. Tahrir, Emilie A. Decoppet, Bianca Cotto, T. Dianne Langford, Shohreh Amini, Kamel Khalili†,

Department of Neuroscience
Center for Neurovirology
Lewis Katz School of Medicine at Temple University
3500 N. Broad Street
Philadelphia, PA 19140

†Co-Corresponding authors
Phone: 215.707.4500
Email: taha.mohseni.ahooyi@temple.edu

A

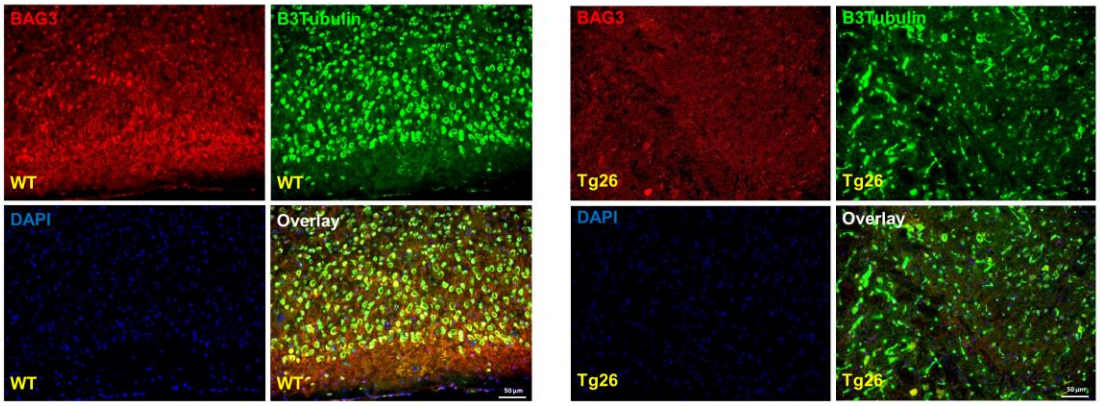


B

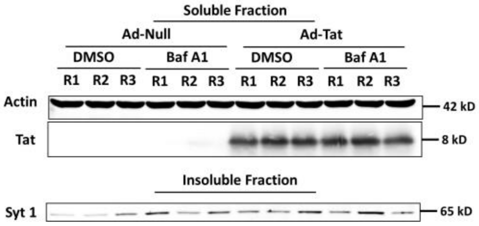


**Fig S1: A.** Immunohistochemistry images indicate BAG3 downregulation in the brain slices from Tg26 mice. **B.** Ad-Tat transduction of primary neurons causes accumulation of Syt1 in insoluble neuronal proteins fraction.

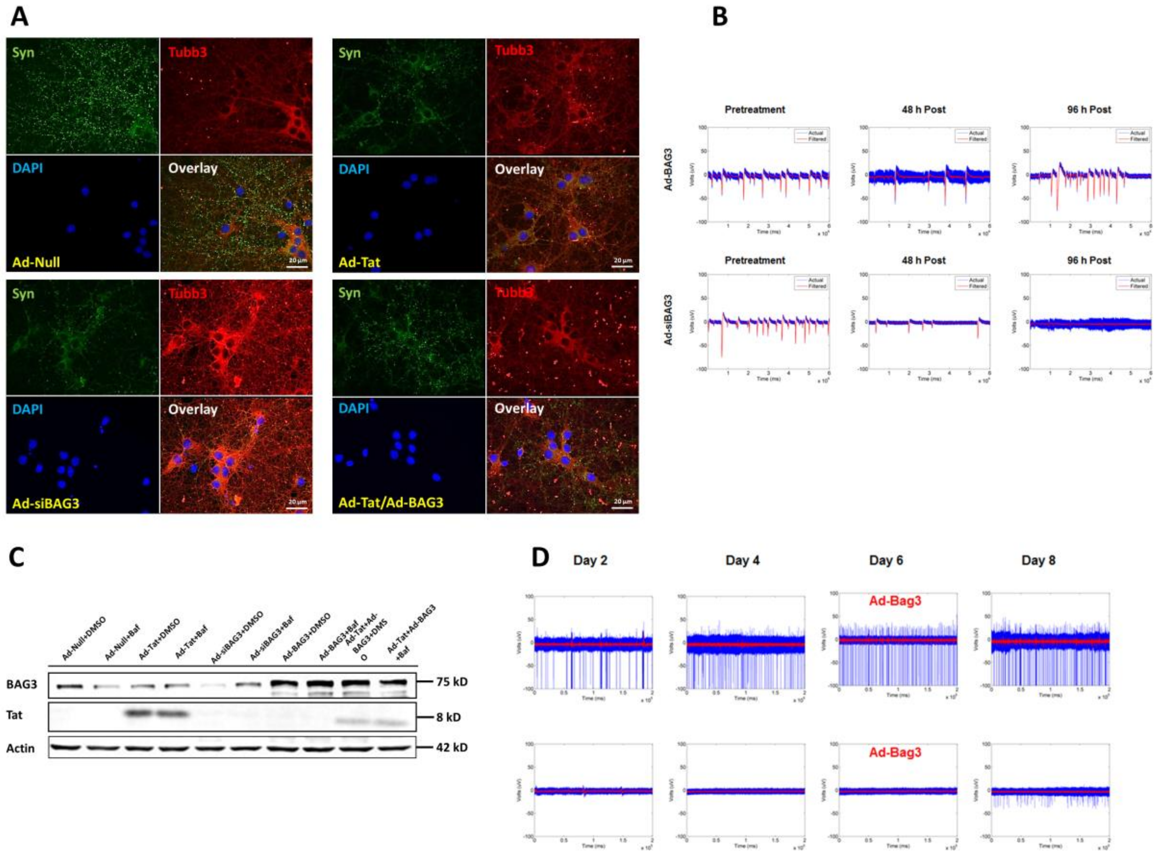


**Figure S2: BAG3 overexpression partially restores synaptic vesicles distribution and neuronal activity. A.** BAG3 overexpression restores the impairing effect of HIV-1 Tat on SVs distribution in immuncytochemical images as probed with synapsin antibody. **B.** BAG3 overexpression lowers Tat expression in neurons. **C and D.** BAG3 overexpression restores the neuronal activity as measured by microelectrode arrays.

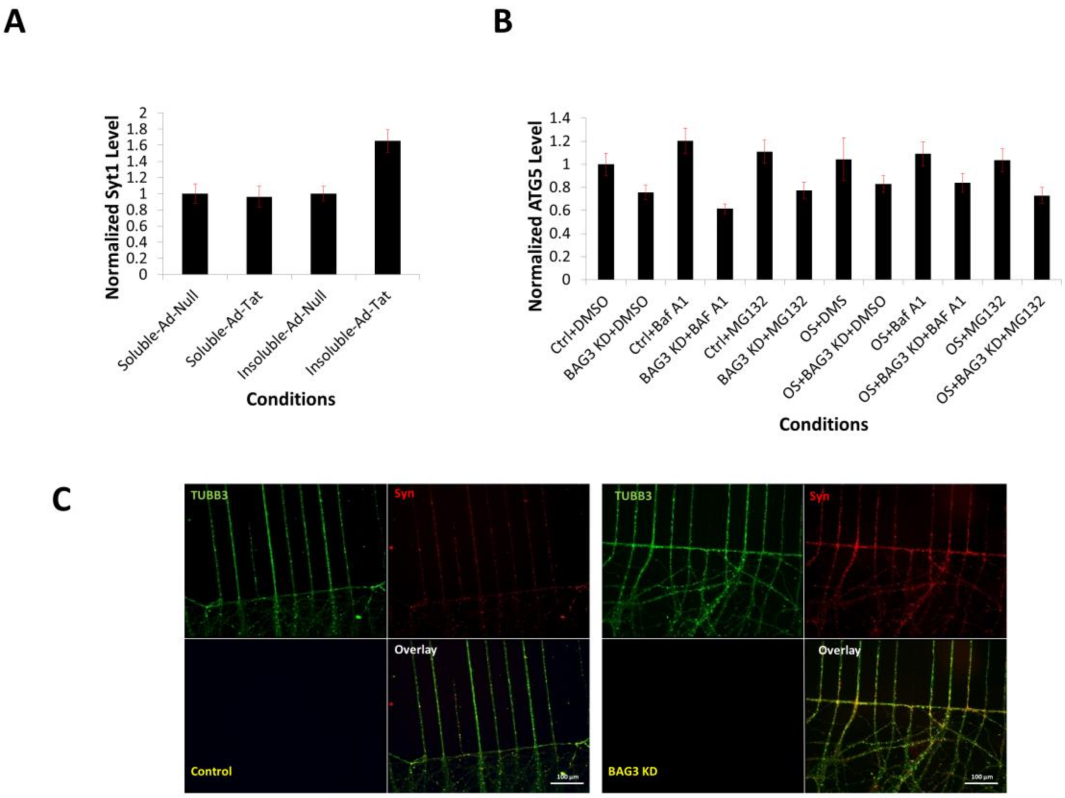


**Figure S3: A.** Syt1 protein levels in the insoluble fraction increased as a result of Tat expression in primary rat neurons. **B.** ATG5 protein levels in primary rat decreased under BAG3 KD in the presence and absence of protein degradation inhibitors and oxidative stress. **C.** BAG3 KD leads to the accumulation of synapsin along axon grown in microfluidic channels.
